# Iodine Status during Pregnancy in a Region of Mild-to-Moderate Iodine Deficiency is not Associated with Adverse Obstetric Outcomes; Results from the Avon Longitudinal Study of Parents and Children (ALSPAC)

**DOI:** 10.3390/nu10030291

**Published:** 2018-03-01

**Authors:** Barbara Torlinska, Sarah C. Bath, Aisha Janjua, Kristien Boelaert, Shiao-Yng Chan

**Affiliations:** 1 Institute of Applied Health Research, University of Birmingham, Birmingham B15 2TT, UK; b.torlinska@bham.ac.uk; 2 Department of Nutritional Sciences, Faculty of Health and Medical Sciences, University of Surrey, Guildford, Surrey GU2 7XH, UK; 3 Birmingham Heartlands Hospital, Birmingham B9 5SS, UK; aishajanjua@doctors.org.uk; 4 Warwick Medical School, University of Warwick, Coventry CV4 7AL, UK; 5 Institute of Metabolism and Systems Research, University of Birmingham, Birmingham B15 2TT, UK; k.boelaert@bham.ac.uk; 6 Centre for Endocrinology, Diabetes and Metabolism, Birmingham Health Partners, Edgbaston, Birmingham B15 2TH, UK; 7 Department of Obstetrics and Gynaecology, Yong Loo Lin School of Medicine, National University of Singapore, 1E Kent Ridge Road, Singapore 119228, Singapore; obgchan@nus.edu.sg

**Keywords:** iodine, pregnancy, obstetric, UK, Avon Longitudinal Study of Parents and Children (ALSPAC)

## Abstract

Severe iodine deficiency during pregnancy has been associated with pregnancy/neonatal loss, and adverse pregnancy outcomes; however, the impact of mild–to–moderate iodine insufficiency, though prevalent in pregnancy, is not well-documented. We assessed whether mild iodine deficiency during pregnancy was associated with pregnancy/infant loss, or with other adverse pregnancy outcomes. We used samples and data from the Avon Longitudinal Study of Parents and Children (ALSPAC), from 3140 singleton pregnancies and from a further 42 women with pregnancy/infant loss. The group was classified as mildly-to-moderately iodine deficient with a median urinary iodine concentration of 95.3 µg/L (IQR 57.0–153.0; median urinary iodine-to-creatinine ratio (UI/Creat) 124 µg/g, IQR 82–198). The likelihood of pregnancy/infant loss was not different across four UI/Creat groups (<50, 50–149, 150–250, >250 µg/g). The incidence of pre-eclampsia, non-proteinuric gestational hypertension, gestational diabetes, glycosuria, anaemia, post-partum haemorrhage, preterm delivery, mode of delivery, being small for gestational age, and large for gestational age did not differ significantly among UI/Creat groups, nor were there any significant differences in the median UI/Creat. We conclude that maternal iodine status was not associated with adverse pregnancy outcomes in a mildly-to-moderately iodine-deficient pregnant population. However, in view of the low number of women with pregnancy/infant loss in our study, further research is required.

## 1. Introduction

Iodine is essential for the production of thyroid hormones and is particularly important during pregnancy and early life owing to its role in brain development. While there has been enormous progress in the eradication of iodine deficiency in many countries worldwide (through the use of iodised salt), iodine deficiency remains a problem in pregnant women in many countries, including the UK [1]. The World Health Organization (WHO) categorises iodine adequacy in a pregnant population if the median urinary iodine concentration (UIC) is 150–249 µg/L; a median UIC in pregnancy of <150 µg/L is considered insufficient, 250–499 µg/L as above requirements and >500 µg/L as excessive [2].

Significant neurodevelopmental impairment occurs with severe maternal iodine deficiency during pregnancy, including the development of cretinism [3]. Correction of severe iodine deficiency in the population decreases the rates of cretinism [4] and increases offspring IQ [5]. More recently, observational studies have found associations between mild-to-moderate iodine deficiency during pregnancy and poorer offspring cognition, IQ, and school performance [6,7,8,9,10].

Beyond the effects on brain development, severe maternal iodine deficiency has also been associated with increased perinatal and infant mortality, recurrent miscarriage and preterm delivery, and significant improvements were observed with iodine supplementation [11,12]. Whether mild iodine deficiency or excess iodine intake can adversely affect pregnancy outcomes is less clear. One retrospective study in Senegal suggested a dose-dependent relationship between the degree of iodine deficiency and the risk of pregnancy loss but this study was subject to recall bias [13]. A prospective observational study reporting outcomes of 390 pregnancies in a mildly iodine-deficient population in Thailand reported increased risks of preterm birth and low birthweight in those with iodine insufficiency compared with those who were iodine sufficient, but there was no significant difference in stillbirth rates [14]. A randomised controlled trial was conducted in India and Thailand evaluating iodine supplementation starting from the end of the first trimester of pregnancy with the primary outcomes of change in maternal thyroid function and child neurodevelopment. It reported no significant change in rates of preterm birth or low birth weight; however, it was underpowered to detect differences in obstetric outcomes, and perinatal losses were not specifically reported [15]. Furthermore, the women in the trial were quite likely to be iodine sufficient, especially in India where the baseline iodine status was in the adequate range [16].

Even women living in countries classed as iodine replete are commonly iodine deficient in pregnancy [1] because of physiologically increased iodine requirements in pregnancy due to increased renal clearance, increased thyroid hormone synthesis and fetal-placental requirements [17,18,19]. With the success of population-based iodine supplementation programmes and with additional routine oral iodine supplementation in pregnancy advocated in parts of the world [20,21], regardless of the iodine status of the population the potential issues associated with iodine excess in pregnancy have also recently surfaced in some countries, including adverse effects on maternal thyroid function [22]. Thus, it is important to establish if mild derangements in iodine status during pregnancy, both insufficiency and iodine excess, is associated with adverse obstetric outcomes. The implications of mild iodine deficiency are of relevance to the UK as studies have reported mild-to-moderate iodine deficiency in teenage schoolgirls [23] and in pregnant women [24,25,26,27].

This study used samples and data from pregnancies in the UK Avon Longitudinal Study of Parents and Children (ALSPAC) birth cohort, where we have previously documented an adverse impact of maternal iodine insufficiency on child neurocognitive development [6]. In this study, we investigated whether insufficient and excess iodine status during pregnancy was associated with adverse obstetric outcomes defined as: (i) pregnancy loss or infant loss by the age of 1 year; (ii) common obstetric complications including hypertensive disorders of pregnancy, glucose derangements, anaemia, preterm delivery, caesarean delivery, post-partum haemorrhage and babies born small or large for gestational age.

## 2. Materials and Methods

ALSPAC recruited pregnant women from the county of Avon in the south-west of the UK, with expected dates of delivery 1 April 1991 to 31 December 1992 [28,29]. Among the 14,541 pregnant women enrolled; there were 553 miscarriages, perinatal losses and infant deaths, and 13,988 children survived for at least 12 months. Ethical approval for the study was obtained from the ALSPAC Ethics and Law Committee and the local research ethics committees [30]. Please note that the study website contains details of all the data that is available through a fully searchable data dictionary [31].

For the current study, women were selected on the basis of a singleton pregnancy and if they had at least one existing measure of urinary iodine-to-creatinine (UI/Creat) during pregnancy; these existing measures were from previous and ongoing studies examining the relationship between maternal iodine status and child IQ at age 8 years and the measures were already available in the ALSPAC resource (*n* = 3524). Furthermore, we selected women who experienced pregnancy/infant loss up to the age of 1 year and who had an available antenatal urine sample for iodine analysis (*n* = 46). In all cases where there was a repeat urine sample, the earliest available sample was used; this was done as we postulated that the impact of iodine status on pregnancy outcome is greatest during early pregnancy.

### 2.1. Laboratory Analysis

Iodine concentration was measured in the urine samples from women with fetal/infant loss up to 1 year in the same laboratory (Trace Element Unit, Southampton General Hospital, Southampton, UK) and using the same methodology (i.e., using inductively coupled plasma mass spectrometer (ICP-MS)) as was used for all previous measures of iodine concentration in ALSPAC urine samples (*n* = 3524); the details of this method have been described in full previously [6]. The results were verified for accuracy using certified reference material, Seronorm Trace Elements Urine (Nycomed Pharma, Norway; with a certified iodine content of 84 µg/L and 305 µg/L for Seronorm Urine Level 1 and 2 respectively). The within run precision was 7.0% at 84 µg/L and 5.8% at 294 µg/L. The between run precision was 4.4% at 43 µg/L, 4.9% at 83 µg/L, 3.6% at 149 µg/L, and 2.3% at 294 µg/L. As previously, urinary creatinine was determined by the UniCel DxC Synchron Clinical System Analyzer (Beckman Coulter, High Wycombe, UK) using the Jaffe rate method.

### 2.2. Classification of Iodine Status

The median urinary iodine concentration (UIC) value of the group was compared with the WHO UIC cut-offs for iodine adequacy in pregnancy [2]. As UIC cannot be used to estimate iodine status in an individual (as a result of day-to-day variation and urine dilution), we used the iodine-to-creatinine ratio (UI/Creat) for analyses that related iodine status to pregnancy outcomes. This is because the UI/Creat corrects for some of the variation in dilution among spot-urine samples; the iodine-to-creatinine ratio produces a better measure of individual iodine status, especially when the age and sex of the individual is taken into account [32], as is the case in a cohort of pregnant women.

We report iodine status in two ways, as the iodine concentration (UIC) in µg/L and as the iodine-to-creatinine ratio (UI/Creat) in µg/g. As in our previous research on iodine status in the ALSPAC cohort, we grouped women according to their UI/Creat result. The groups were defined as <50 µg/g, 50–149.9 µg/g, 150–249.9 µg/g, and ≥250 µg/g; these groups, broadly speaking relate to severely deficient, mildly-to-moderately deficient, sufficient, and more-than adequate iodine, respectively, based on WHO [2] and other published criteria [3]. We investigated the relationship between UI/Creat during pregnancy with obstetric outcomes in women who had infants alive at 1 year by comparing the incidence of common pregnancy complications in the four iodine status categories.

### 2.3. Obstetric Outcomes

We examined the following obstetric outcomes: (i) hypertensive disorders in pregnancy; (ii) glucose derangement; (iii) anaemia; (iv) post-partum haemorrhage; (v) preterm birth; (vi) mode of delivery; and (vii) birthweight. Details of obstetric outcomes were extracted from hospital records following delivery. Hypertensive disorders in pregnancy was defined by a systolic BP > 139 mmHg or a diastolic BP > 89 mmHg on 2 or more occasions beyond 20 weeks gestation, as previously defined in this cohort [33], and included two subgroups: pre-eclampsia (at least 1+ proteinuria with episode of hypertension) and non-proteinuric gestational hypertension. We examined any form of glucose derangement in pregnancy, including: (i) gestational diabetes (diagnosis obtained from medical records in women with no history of pre-existing diabetes); (ii) glycosuria (dipstick glycosuria of ++ or more on at least two occasions in women with no evidence of pre-existing or gestational diabetes); and (iii) hyperglycaemia (including cases of any abnormality in fasting bloods, serial blood sugars, glucose-tolerance tests or fructosamine evaluations). Anaemia was defined by a haemoglobin of between 4.0 g/dL and 9.9 g/dL. Post-partum haemorrhage was defined by an estimated blood loss of over 500 mL around the time of delivery. Gestational age was determined by the last menstrual period but adjusted if early pregnancy ultrasound measurements differed by 2 weeks or more, according to the clinical protocol in use at the time. Preterm delivery was defined as birth before 37 completed weeks of pregnancy, including both spontaneous onset of labour and iatrogenic deliveries. All caesarean section deliveries were considered as a single group whilst vaginal deliveries were subdivided into spontaneous or instrumental/assisted/breech deliveries. Birthweights were customised for sex, gestational age, maternal body mass index (BMI), parity and ethnicity as previously described [34] to identify babies who were small for gestational age (<10th percentile) and large for gestational age (>90th percentile). The outcomes were analysed within the subsets of available data for each individual outcome. No data imputation was conducted.

### 2.4. Statistical Analysis

As a result of concern that the urine samples had been contaminated with iodine from urine test strips [6], women with excessively high urinary iodine concentration (>500 mg/L) and/or high iodine-to-creatinine ratio (>700 mg/g) were excluded from statistical analysis (those with a child alive at 1 year: *n* = 414, 11.7%; those with pregnancy/infant loss *n* = 5, 10.9%). If a woman had a later urine sample (with a result that was considered to be uncontaminated) that was used to replace the contaminated result (those with a child alive at 1 year *n* = 50; those with pregnancy/infant loss *n* = 1). We also excluded women who were taking thyroid hormone medication during pregnancy (those with a child alive at 1 year *n* = 20, 0.5%; those with pregnancy/infant loss, *n* = 0, 0%). This resulted in a total of 3140 women with a child alive at 1 year, and 42 women with a pregnancy/infant loss.

UI/Creat was not normally distributed and, therefore, differences between groups were compared using a non-parametric test (Mann–Whitney U test). Additionally, UI/Creat were stratified into four iodine status groups as described above. Chi-square tests were used when comparing groups of iodine status with the presence/absence of pregnancy outcomes, and one-way analysis of variance (ANOVA) was used to compare continuous data between grouped UI/Creat.

Likelihood of fetal and neonatal death events was compared to live controls. Adverse pregnancy outcomes were studied within those with live babies. Logistic regression was used to examine the association between maternal iodine status (as the four groups of UI/Creat) and likelihood of adverse pregnancy outcomes, with women in the 150–249 µg/g group as the reference category. For each pregnancy outcome, crude and adjusted odds ratios of the pregnancy outcomes (OR and AOR) were calculated. The likelihood of being small or large for gestational age were calculated against odds of being appropriately grown for gestational age. The models were adjusted for mother’s BMI (pre-pregnancy) and age, parity (zero, one, two, three or more), cigarette smoking during early pregnancy (yes/no) and trimester of UI/Creat collection.

The statistical significance was assumed a priori at 0.05 level; the 95% confidence intervals (CI) were calculated accordingly. All analyses were conducted using IBM SPSS Statistics version 23 (IBM Corp., Armonk, NY, USA).

## 3. Results

In total we included 42 cases of pregnancy/infant loss up to age 1 year. This included fetal deaths and stillbirths between 20–42 weeks’ gestation (*n* = 11), neonatal deaths within the first month of life (*n* = 24), and child deaths between 1–12 months (*n* = 7). We also had uncontaminated UI/Creat results from a total of 3140 women with babies who survived beyond the first year.

The median UIC in the entire cohort of women included in this study (*n* = 3182) was 95.3 µg/L (IQR 57.0–153.0), indicating mild-to-moderate iodine deficiency in this group of pregnant women [35]. The median (IQR) urinary iodine-to-creatinine (UI/Creat) was 124 (82–198) µg/g. The UI/Creat was <50 µg/g in 5.1% (*n* = 161) of the cohort, 50–149.9 µg/g in 56.4% (*n* = 1794), 150–249.9 µg/g in 21.8% (*n* = 693) and greater than 250 µg/g in 16.8% (*n* = 534).

### 3.1. Iodine Status and Pregnancy Loss

We compared women with infants alive at 1 year (*n* = 3140) to those with pregnancy/infant loss (*n* = 42) (Table 1). There were no significant differences in maternal BMI, age, parity and sex of the baby. Furthermore, there were no differences in other potential confounders, such as history of miscarriage and cigarette smoking. However, the urine samples used for UI/Creat measurements were collected significantly earlier in gestation in the group with a live infant compared with the pregnancy/infant loss group (*p* < 0.001). The median UI/Creat of those with pregnancy/infant loss and those with babies alive at 1 year was not significantly different (144 vs. 123 µg/g; *p* = 0.07).

Amongst those who experienced a pregnancy/child loss, the likelihood of being iodine insufficient or more-than adequate during the pregnancy were no different to those whose child was alive at one year. Adjustment for confounders (trimester of urine collection, BMI, age, parity, history of miscarriage and smoking) did not alter the conclusions.

### 3.2. Iodine Status and Pregnancy Outcomes among Women with Infants Alive at One Year

There were significant differences between the four UI/Creat status groups in terms of BMI, age, parity and trimester of UI/Creat collection (Table 2). The <50 µg/g group was associated with a higher BMI, younger age, lower parity and urinary collection earlier in pregnancy.

The overall incidence and the odds of hypertensive disorders in pregnancy and the subgroups of pre-eclampsia and non-proteinuric gestational hypertension were not significantly different between the four groups of iodine status (Table 3). Similarly, the overall incidence and odds of any form of glucose derangement in pregnancy, as well as the subgroups of gestational diabetes, glycosuria and hyperglycaemia in pregnancy were no different between the iodine status groups. The incidence and odds of anaemia both antenatally and during the first 14 days post-partum as well as the incidence of post-partum haemorrhage were not different either. There were no differences in preterm delivery rates or in the mode of delivery between groups. Of the 1954 cases where customised birthweight percentiles [34] could be calculated, the mean (SD) percentiles were similar across the groups (46.3% (29.5) with UI/Creat less than 50 µg/g (*n* = 95), 50.7% (28.9) with UI/Creat 50–149.9 µg/g (*n* = 1132), 48.7% (28.4) with UI/Creat 150–249.9 µg/g (*n* = 392) and 46.9% (29.1) with UI/Creat greater than 250 µg/g (*n* = 335)) and the incidence and odds of being both small for gestational age and large for gestational age were not significantly different between iodine groups. We also modelled the likelihood (odds ratio) of adverse pregnancy outcomes in various UI/Creat status groups compared with sufficient UI/Creat, adjusting for the confounders of mothers’ BMI, age, parity, smoking and trimester of UI/Creat collection (Table 3). There were no significant differences in the adjusted odds ratios for any of the outcomes.

Additionally, we compared the UI/Creat as a continuous variable in those with and without a pregnancy complication of interest. There were no significant differences in the median UI/Creat when women were dichotomised based on the presence or absence of the studied outcome (Table 4).

We performed a sensitivity analysis limited to cases where the UI/Creat ratio was obtained from a first trimester sample. Similar to the entire cohort, there were significant differences in BMI, parity and age of women across four UI/Creat categories (Supplementary Table S1). No differences were observed in the incidence of adverse pregnancy outcomes (Supplementary Table S2) except for hyperglycaemia during pregnancy being more likely in mothers with UIC/Creat ≥ 250 µg/g compared with those in 150–249 µg/g group (AOR = 2.5 (1.1–5.5), *p* = 0.03), which is most likely a chance finding (Type 1 error). There were no significant differences in the median UIC/Creat between the groups with and without a pregnancy complication of interest (Supplementary Table S3).

## 4. Discussion

This is one of the first cohort studies in an overall mildly-to-moderately iodine-deficient pregnant population to document that iodine insufficiency and more-than-adequate iodine status is not associated with pregnancy or infant loss up to 1 year of age, or with other adverse obstetric outcomes.

It has been proposed that the potential adverse effects of severe iodine deficiency in pregnancy are mediated through maternal thyroid dysfunction as iodine is an essential constituent of thyroid hormones. Indeed, overt hypothyroidism during pregnancy, caused by conditions other than iodine deficiency, has been associated with increased risk of pregnancy loss, preterm delivery, pre-eclampsia, pregnancy-induced hypertension, low birthweight, caesarean delivery, antenatal anaemia and post-partum haemorrhage [36,37]. At an individual level, thyroid function parameters are, however, unreliable indicators of iodine status because of the homeostatic capacity to compensate for mild-to-moderate iodine deficiency [38]; pregnant women can have normal thyroid function even when mildly iodine deficient. The possibility of an alternative mechanism (other than through maternal thyroid dysfunction) which could mediate the potential adverse effects of iodine deficiency upon pregnancy has to be considered. The results of our study show that both mild iodine insufficiency and mild iodine excess are not associated with pregnancy and infant losses, and other obstetric outcomes. This might suggest that maternal and fetal-placental tissues, other than the thyroid and brain, are not particularly sensitive to minor perturbations in iodine availability and can continue to function normally in pregnancy. However, given the low number of pregnancy and infant losses in our study, it is also possible that our study was underpowered to detect the effects of iodine insufficiency.

Many of the previous studies evaluating iodine status in pregnant women living in mildly iodine-deficient countries have reported obstetric outcomes independent of the effects of maternal thyroid function. Consistent with our results on pregnancy/infant loss, a prospective study of a mildly iodine-deficient pregnant population in Thailand using UIC measurements in a smaller cohort [14] reported no association between pregnancy loss and iodine insufficiency, but the study was underpowered to examine this. In our study, we found no significant difference in median iodine-to-creatinine ratio between those with pregnancy/infant loss and those with babies alive at 1 year. Although the median UI/Creat was higher in those with pregnancy/infant loss (though not significantly), this is most likely explained by fact that urine samples were collected significantly later in pregnancy in those with a pregnancy/infant loss; in ALSPAC, as in other UK studies iodine-to-creatinine increases across gestation [25].

We found no association with pregnancy outcomes, which is in contrast to the study in Thailand, where an increase in preterm birth and low birthweight with iodine insufficiency was reported [14]. Birthweight has also been associated with maternal iodine status in a study of mildly deficient pregnant women in Spain, where a lower likelihood of being small for gestational age was reported for women with a UIC in the third trimester of between 100 µg/L and 149 µg/L compared with women with a UIC below 50 µg/L [39]. This is in contrast to our study, where we found no association between iodine status throughout pregnancy and likelihood of being small for gestational age.

The Generation R birth cohort in the Netherlands, which recruited from a pregnant population with optimal iodine status, showed that the subset of women with urinary iodine below the 10th percentile (equivalent to mild iodine insufficiency) were less likely to experience an operative delivery, both instrumental and caesarean section [40]. This is in contrast to our results in the ALSPAC cohort where the lowest assisted delivery rate was actually observed in the iodine-sufficient group (150–249 µg/g), although the difference was not statistically significant. However, consistent with our findings, the Generation R cohort [40] also did not detect any difference in gestational age at delivery nor in birth weight, unlike the Thai cohort [14].

The reasons for these discrepancies are unclear. It may suggest that different confounding factors associated with iodine insufficiency could be at play in influencing obstetric outcomes in different settings. In the absence of good methods which can be used to assess individual iodine status we are limited to assessing iodine status of whole populations, thus the challenge has always been to determine the normal baseline rate of specific obstetric complications in an iodine-sufficient population, and whether the observed complication rate in an iodine-insufficient population could be improved with better iodine status. Even the recently reported clinical trial conducted in India and Thailand, which showed no change in the rates of preterm birth or low birth-weight infants with iodine supplementation in pregnancy, was confounded by the spontaneous improvement in urinary iodine concentration in the placebo group as gestation progressed, such that controls were no longer iodine insufficient by the end of pregnancy [15] and the fact that the women were not very iodine deficient at baseline.

The strength of our study is the relatively large sample size of a pregnant population that is not exposed to any universal iodination programme, and the prospective nature of the study where iodine status was evaluated in a urine sample taken before the occurrence of the adverse events of interest. Because of the sample size we were able to stratify pregnant women into four categories of iodine status for comparison. A limitation of our study is the use of a single time-point spot urinary iodine measure for categorisation of iodine status which may be a poor proxy of individual iodine status [41], so misclassification is possible and could have masked any possible differences between groups. The causes of perinatal and child loss were diverse and the numbers were too small to evaluate if specific causes were more likely to be associated with maternal iodine status. Also, the impact of iodine status on early pregnancy miscarriages could not be studied because urine samples were not collected from these women. Our study is underpowered to detect small differences in outcome incidences and to assess rarer but significant obstetric complications, such as placental abruption. It is also limited in exploring the effects of high iodine status (more-than-adequate or excessive) on pregnancy outcomes as the population of women in ALSPAC were iodine deficient and most women were in the iodine-deficient categories. The majority of participants in ALSPAC were White and had indicators of higher socio-economic position [29] so our findings may not replicate in other ethnic and socioeconomic groups.

## 5. Conclusions

In conclusion, our present study in a mildly-to-moderately iodine-deficient population without an iodised salt programme did not find an association between iodine status and pregnancy outcomes or child loss. Further research in this area is required, given the small number of studies that have evaluated pregnancy outcomes and iodine status, and given the low numbers of women in our study with pregnancy/infant loss.

## Figures and Tables

**Table 1:** Demographic characteristics and urinary iodine-to-creatinine (UI/Creat) measurements in women with a child alive at 1 year and those who experienced pregnancy/infant loss up to 1 year.

	Child Alive at 1 Year *n* = 3140 (98.7%)	Pregnancy/Infant Loss *n* = 42 (1.3%)	*p* Value ^2^
Body mass index (BMI) (kg/m^2^) ^1^	22.25 (20.3–24.5)	21.7 (20.1–24.5)	0.36
Underweight (<18.5)	114 (4.0)	2 (7.7)	0.53
Healthy weight (18.5–24.9)	2129 (74.8)	20 (76.9)	
Overweight (25–29.9)	460 (16.2)	4 (15.4)	
Obese (≥;30)	142 (5.0)	0 (0)	
Age (years), median [IQR]	29 (26–32)	28 (26–31)	0.20
Parity			
0	1428 (47.3)	17 (43.6)	0.27
1	1031 (34.2)	11 (28.2)	
2	426 (14.1)	7 (17.9)	
3+	132 (4.4)	4 (10.3)	
History of miscarriage	598 (19.8)	8 (20.5)	0.91
Cigarette-smoking	1294 (42.2)	16 (41.0)	0.88
Child sex			
Male	1550 (49.4)	26 (61.9)	0.11
Trimester of urine sample			
1st (≤12 weeks)	1735 (55.3)	16 (39.0)	<0.001
2nd (13–27 weeks)	1327 (42.3)	19 (46.3)	
3rd (≥28 weeks)	78 (2.5)	6 (14.6)	
UI/Creat median [IQR] (µg/g)	123 (82–197)	144 (103–209)	0.07
UI/Creat groups			
<50 µg/g	161 (5.1)	0 (0)	0.24
50–149.9 µg/g	1773 (56.5)	21 (50.0)	
150–249.9 µg/g	680 (21.7)	13 (31.0)	
≥250 µg/g	526 (16.8)	8 (19.0)	

^1^ Data are median (25th, 75th percentile) for continuous data and *n* (%) for categorical data. ^2^ *p* value from chi-square test for categorical data and from Mann–Whitney U for continuous data.

**Table 2:** Demographic characteristics across groups categorised by urinary iodine-to-creatinine ratio (UI/Creat) of 3140 pregnant women with babies alive at one year.

	No. Available	UI/Creat Groups (µg/g)				*p* Value ^2^
		<50	50–149.9	150–249.9	≥250	
Age (years)	3122					
*n*		158	1764	675	525	
Mean (SD)		27.2 (4.3)	29.3 (4.5)	29.9 (4.3)	29.9 (4.4)	<0.001
BMI (kg/m^2^) ^1^	2845					
Underweight (<18.5)		6 (4.2)	60 (3.7)	26 (4.2)	22 (4.6)	<0.001
Healthy weight (18.5–24.9)		96 (67.6)	1180 (73.5)	482 (77.6)	371 (77.9)	
Overweight (25–29.9)		25 (17.6)	278 (17.3)	89 (14.3)	68 (14.3)	
Obese (≥30)		15 (10.6)	88 (5.5)	24 (3.9)	15 (3.2)	
Parity ^1^	3017					
0		96 (62.7)	813 (47.7)	285 (43.6)	234 (46.2)	0.007
1		38 (24.8)	591 (34.7)	230 (35.2)	172 (33.9)	
2		17 (11.1)	227 (13.3)	105 (16.1)	77 (15.2)	
3+		2 (1.3)	72 (4.2)	34 (5.2)	24 (4.7)	
Cigarette-smoking ^1^	3067	75 (49.0)	720 (41.7)	287 (43.0)	212 (40.8)	0.30
History of miscarriage ^1^	3022	21 (13.8)	332 (19.4)	140 (21.5)	105 (20.6)	0.18
Child sex ^1^	3140					
Male		67 (41.6)	880 (49.6)	354 (52.1)	249 (47.3)	0.08
Trimester of urine collection ^1^	3140					
1st (≤12 weeks)		122 (75.8)	1114 (62.8)	331 (48.7)	168 (31.9)	
2nd (13–27 weeks)		37 (23.0)	628 (35.4)	325 (47.8)	337 (64.1)	<0.001
3rd (≥28 weeks)		2 (1.2)	31 (1.7)	24 (3.5)	21 (4.0)	

^1^ Values are *n* (%). ^2^ *p* value from chi-squared test for categorical data and analysis of variance (ANOVA) for continuous data.

**Table 3:** Likelihood of each adverse pregnancy outcome in urinary iodine-to-creatinine ratio (UI/Creat) groups; reference group 150–249 µg/g. Data based on 3140 pregnant women with infants alive at one year.

	UI/Creat (µg/g) Group	*n*/*N* ^1^	Incidence		Unadjusted OR		Adjusted OR ^3^	
			(%)	*p* Value ^2^	OR (CI)	*p* Value	AOR (CI)	*p* Value
Hypertensive disorders of pregnancy	<50 50–149 5150–249 5≥250	29/158 277/1757 93/671 87/522	18.4 15.8 13.9 16.7	0.43	1.4 (0.9–2.2) 1.2 (0.9–1.5) 1.0 1.2 (0.9–1.7)	0.15 0.24 0.18	1.2 (0.7–1.9) 1.1 (0.8–1.4) 1.0 1.3 (0.9–1.8)	0.57 0.66 0.17
*Pre-eclampsia*	<50 50–149 150–249 ≥250	2/158 40/1757 10/671 10/522	1.3 2.3 1.5 1.9	0.60	0.8 (0.2–3.9) 1.5 (0.8–3.1) 1.0 1.3 (0.5–3.1)	0.83 0.23 0.57	0.6 (0.1–3.1) 1.2 (0.6–2.6) 1.0 1.5 (0.6–3.7)	0.57 0.61 0.41
*Non-proteinuric gestational hypertension*	<50 50–149 150–249 ≥250	27/158 237/1757 83/671 77/522	17.1 13.5 12.4 14.8	0.38	1.5 (0.9–2.3) 1.1 (0.8–1.4) 1.0 1.2 (0.9–1.7)	0.12 0.47 0.23	1.2 (0.7–2.1) 1.0 (0.8–1.4) 1.0 1.2 (0.9–1.8)	0.39 0.82 0.26
Any glucose derangement ^4^	<50 50–149 150–249 ≥250	12/94 124/1116 41/395 43/333	12.8 11.1 10.4 12.9	0.70	1.3 (0.6–2.5) 1.1 (0.7–1.6) 1.0 1.3 (0.8–2.0)	0.50 0.69 0.29	1.1 (0.5–2.3) 1.0 (0.7–1.5) 1.0 1.4 (0.9–2.3)	0.79 0.97 0.17
*Gestational diabetes*	<50 50–149 150–249 ≥250	1/154 7/1699 3/654 7/514	0.6 0.4 0.5 1.4	0.10	1.4 (0.1–13.7) 0.9 (0.2–3.5) 1.0 3.0 (0.8–11.6)	0.76 0.88 0.11	1.6 (0.2–17.1) 0.5 (0.1–2.3) 1.0 3.3 (0.8–13.1)	0.69 0.36 0.15
*Glycosuria* *(2+ or more)*	<50 50–149 150–249 ≥250	5/158 53/1757 28/671 22/522	3.2 3.0 4.2 4.2	0.40	0.8 (0.3–2.0) 0.7 (0.4–1.1) 1.0 1.0 (0.6–1.8)	0.56 0.16 0.97	0.7 (0.2–2.0) 0.7 (0.4–1.2) 1.0 0.9 (0.5–1.8)	0.46 0.18 0.84
*Hyperglycaemia during pregnancy*	<50 50–149 150–249 ≥250	9/96 115/1149 34/402 42/339	9.4 10.0 8.5 12.4	0.36	1.1 (0.5–2.4) 1.2 (0.8–2.5) 1.0 1.5 (0.9–2.5)	0.77 0.36 0.08	1.0 (0.5–2.4) 1.1 (0.7–1.7) 1.0 1.6 (1.0–2.7)	0.91 0.69 0.07
Anaemia during pregnancy	<50 50–149 150–249 ≥250	10/96 123/1149 47/402 34/339	10.4 10.7 11.7 10.0	0.90	0.9 (0.4–1.8) 0.9 (0.6–1.3) 1.0 0.8 (0.5–1.3)	0.73 0.59 0.47	1.2 (0.5–2.5) 1.0 (0.6–1.4) 1.0 0.9 (0.6–1.5)	0.69 0.81 0.75
Anaemia < 14 days postpartum	<50 50–149 150–249 ≥250	17/158 224/1755 78/671 53/522	10.8 12.8 11.6 10.2	0.40	0.9 (0.5–1.6) 1.1 (0.8–1.5) 1.0 0.9 (0.6–1.2)	0.76 0.45 0.42	0.6 (0.3–1.1) 1.0 (0.7–1.4) 1.0 0.8 (0.6–1.2)	0.11 0.91 0.33
Postpartum haemorrhage	<50 50–149 150–249 250+	15/95 164/1133 46/387 49/336	15.8 14.5 11.9 14.6	0.58	1.4 (0.7–2.6) 1.3 (0.9–1.8) 1.0 1.3 (0.8–1.9)	0.31 0.20 0.29	1.2 (0.6–2.3) 1.2 (0.8–1.7) 1.0 1.3 (0.8–2.0)	0.61 0.46 0.34
Preterm delivery	<50 50–149 150–249 ≥250	9/158 80/1764 29/675 28/525	5.7 4.5 4.3 5.3	0.76	1.3 (0.6–2.9) 1.1 (0.7–1.6) 1.0 1.3 (0.7–2.1)	0.45 0.80 0.40	1.3 (0.6–3.0) 1.0 (0.6–1.6) 1.0 1.4 (0.8–2.4)	0.48 0.98 0.29
Caesarean section	<50 50–149 150–249 ≥250	14/95 192/1113 63/386 61/335	14.7 17.3 16.3 18.2	0.84	0.9 (0.5–1.7) 1.1 (0.8–1.5) 1.0 1.1 (0.8–1.7)	0.71 0.68 0.50	0.6 (0.3–1.3) 0.9 (0.7–1.3) 1.0 1.0 (0.7–1.6)	0.22 0.66 0.88
Assisted/breech delivery among all vaginal births	<50 50–149 150–249 ≥250	23/81 231/921 67/323 72/274	28.4 25.1 20.1 26.3	0.70	1.2 (0.7–2.2) 1.3 (0.9–1.8) 1.0 1.3 (0.9–1.9)	0.50 0.12 0.18	1.2 (0.7–2.3) 1.1 (0.8–1.6) 1.0 1.0 (0.7–1.6)	0.50 0.45 0.93
Small for gestational age ^4^	<50 50–149 150–249 ≥250	13/95 111/1132 36/392 43/335	13.7 9.8 9.2 12.8	0.23	1.6 (0.8–3.0) 1.1 (0.7–1.6) 1.0 1.5 (0.9–2.3)	0.19 0.72 0.12	1.5 (0.7–3.1) 1.0 (0.7–1.6) 1.0 1.5 (0.9–2.5)	0.29 0.87 0.13
Large for gestational age ^4^	<50 50–149 150–249 ≥250	5/95 124/1132 30/392 26/335	5.3 11.0 7.7 7.8	0.06	0.7 (0.3–1.8) 1.5 (1.0–2.3) 1.0 1.0 (0.6–1.8)	0.42 0.06 0.96	0.7 (0.2–1.8) 1.4 (0.9–2.2) 1.0 1.3 (0.6–1.9)	0.66 0.12 0.80

^1^ Values are number affected/total in the category (*n*/*N*). ^2^ *p* value from Chi square. ^3^ Logistic regression model adjusted for mother’s BMI and age, parity, cigarette smoking, and trimester of UI/Creat collection. ^4^ Customized for maternal BMI, parity, sex and gestational age [34]; likelihood of being small or large for gestational age were calculated against odds of being appropriately grown for gestational age.

**Table 4:** Comparison of urinary iodine-to-creatinine ratio (UI/Creat; µg/g) in those where pregnancy complications were either present or absent. Data based on 3140 pregnant women with infants alive at one year.

	Median Urinary Iodine-to-Creatinine Ratio (µg/g) ^1^				*p* Value ^2^
	*n*	Complication Present	*n*	Complication Absent	
**Hypertensive disorders of pregnancy**	486	119 (80–199)	2622	124 (82–197)	0.63
*Pre-eclampsia*	62	117 (89–192)	3046	124 (82–198)	0.86
*Non-proteinuric gestational hypertension*	424	121 (79–202)	2684	124 (83–197)	0.55
**Any glucose derangement**	220	124 (78–210)	1718	123 (83–192)	0.89
*Gestational diabetes*	18	170 (95–357)	3003	124 (82–197)	0.14
*Glycosuria (2 + or more)*	108	140 (8–219)	3000	123 (82–197)	0.27
*Hyperglycaemia during pregnancy*	200	123 (78–217)	1786	123 (82–192)	0.94
**Anaemia during pregnancy**	214	123(91–202)	1772	123 (81–193)	0.35
**Anaemia < 14 days postpartum**	372	119 (82–190)	2734	124 (82–199)	0.27
**Postpartum haemorrhage**	274	116 (79–192)	1677	124 (82–195)	0.34
**Preterm delivery**	146	122 (82–197)	2976	124 (82–198)	0.92
**Mode of delivery**	**Caesarean section**		**Vaginal delivery**		
	330	125 (84–203)	1599	123(81–196)	0.53
**Type of vaginal delivery**	**Assisted/breech**		**Spontaneous**		
	393	122 (78–195)	1206	123 (82–196)	0.55
**Customised birthweight ^3^**	**Small for gestational age**		**Adequately grown for gestational age**		
	203	125 (80–207)	1566	124 (82–198)	0.89
	**Large for gestational age**		**Adequately grown for gestational age**		
	185	115 (79–164)	1566	124 (82–198)	0.14

^1^ Values are median (25th, 75th percentile). ^2^ *p* value from Mann–Whitney U non-parametric tests. ^3^ Customized for maternal BMI, parity, sex and gestational age [34].

## Supplementary Materials

The following are available online at http://www.mdpi.com/2072-6643/10/3/291/s1, Table S1: Demographic characteristics according to grouped urinary iodine-to-creatinine ratio (UI/Creat) from the first trimester only, Table S2: Likelihood of each adverse pregnancy outcome in urine-to-creatinine (UI/Creat) group for first trimester samples only, Table S3: Comparison of urinary iodine-to-creatinine ratio (UI/Creat; µg/g) in the first trimester only in those where pregnancy complications were either present or absent.
