# Supplementary material for: Iodine Status during Pregnancy in a Region of Mild-to-Moderate Iodine Deficiency is not Associated with Adverse Obstetric Outcomes; Results from the Avon Longitudinal Study of Parents and Children (ALSPAC)
Source: Nutrients. 2018 Mar 1;10(3):291. doi: 10.3390/nu10030291 (PMC5872709; doi:10.3390/nu10030291)
Supplement: Supplementary file 1 [file nutrients-10-00291-s001.docx]

**Supplementary Materials**

**Table S1.** Demographic characteristics according to grouped urinary iodine-to-creatinine ratio (UI/Creat) from the first trimester only.

|  | **No. Available** | **UI/Creat Groups (µg/g)** | | | | ***p* Value** |
| --- | --- | --- | --- | --- | --- | --- |
|  |  | <50.0 | 50.0–149.9 | 150.0–249.9 | ≥ 250.0 |  |
| Age(years) | 1735 |  |  |  |  |  |
| *n* |  | 122 | 1114 | 331 | 168 |  |
| mean (SD) |  | 27.4 (4.5) | 29.1 (4.4) | 29.9 (4.3) | 31.0 (4.5) | <0.001 |
| Body mass index (BMI) (kg/m^2^) ^1^ | 1579 |  |  |  |  |  |
| *Underweight (<18.5)* |  | 4 (3.6) | 35 (3.5) | 13 (4.3) | 8 (5.2) | 0.02 |
| *Healthy weight (18.5–24.9)* |  | 71 (64.5) | 744 (73.5) | 238 (78.8) | 117 (75.5) |  |
| *Overweight (25–29.9)* |  | 22 (20.0) | 178 (17.6) | 43 (14.2) | 25 (16.1) |  |
| *Obese (>30)* |  | 13 (11.8) | 55 (5.4) | 8 (2.6) | 5 (3.2) |  |
| Parity ^1^ | 1674 |  |  |  |  |  |
| *0* |  | 73 (62.4) | 507 (47.3) | 143 (44.8) | 70 (42.2) | 0.03 |
| *1* |  | 28 (23.9) | 381 (35.5) | 118 (35.5) | 56 (33.7) |  |
| *2* |  | 14 (12.0) | 142 (13.2) | 44 (13.8) | 34 (20.5) |  |
| *3+* |  | 2 (1.7) | 42 (3.9) | 14 (4.4) | 6 (3.6) |  |
| Cigarette-smoking ^1^ | 1693 | 58 (49.6) | 449 (41.4) | 131 (40.4) | 63 (37.5) | 0.22 |
| History of miscarriage ^1^ | 1671 | 18 (15.5) | 203(18.9) | 64(20.2) | 36 (2.20) | 0.56 |
| Child sex ^1^ | 1735 |  |  |  |  |  |
| *Male* |  | 51 (41.8) | 571 (51.3) | 176 (53.2) | 72 (42.9) | 0.03 |

1. Values are n (%). 2. *p* value from chi-square test for categorical data and analysis of variance (ANOVA) for continuous data.

**Table S2.** Likelihood of each adverse pregnancy outcome in urine-to-creatinine (UI/Creat) group for first trimester samples only. Reference group 150–249 µg/g.

|  | **Iodine-to-Creatinine (µg/g)** | **n/N ^1^** | **Incidence** | |
| --- | --- | --- | --- | --- |
|  |  |  | **(%)** | ***p* Value ^2^** |
| Hypertensive disorders of pregnancy | <50.0  50–149  150–249  ≥250 | 17/122  174/1104  42/326  31/167 | 13.9  15.8  12.9  18.6 | 0.36 |
| *Pre–eclampsia* | <50.0  50–149  150–249  ≥250 | 1/122  25/1104  4/326  4/167 | 0.8  2.3  1.2  2.4 | 0.49 |
| *Non–proteinuric gestational hypertension* | <50.0  50–149  150–249  ≥250 | 16/122  149/1104  38/326  27/167 | 13.1  13.5  11.7  16.2 | 0.58 |
| Any glucose derangement ^3^ | <50.0  50–149  150–249  ≥250 | 11/72  78/705  16/193  17/99 | 15.3  11.1  8.3  17.2 | 0.10 |
| *Gestational diabetes* | <50.0  50–149  150–249  ≥250 | 1/118  4/1069  1/319  1/165 | 0.8  0.4  0.3  0.6 | 0.85 |
| *Glycosuria (2+ or more)* | <50.0  50–149  150–249  ≥250 | 4/122  30/1104  11/326  8/167 | 3.3  2.7  3.4  4.8 | 0.53 |
| *Hyperglycaemia during pregnancy* | <50.0  50–149  150–249  ≥250 | 8/74  77/725  13/197  17/100 | 10.8  10.6  6.6  17.0 | 0.05 |
| Anaemia during pregnancy | <50.0  50–149  150–249  ≥250 | 6/74  70/725  25/197  9/100 | 8.1  9.7  12.7  9.0 | 0.56 |
| Anaemia <14 days postpartum | <50.0  50–149  150–249  ≥250 | 10/122  135/1103  41/326  18/167 | 8.2  12.2  12.6  10.8 | 0.56 |
| Postpartum haemorrhage | <50.0  50–149  150–249  ≥250 | 10/73  104/717  23/190  17/97 | 13.7  14.5  12.1  17.5 | 0.66 |
| Preterm delivery | <50.0  50–149  150–249  ≥250 | 4/122  47/1110  17/327  11/168 | 3.3  4.2  5.2  6.5 | 0.47 |
| Caesarean section | <50.0  50–149  150–249  ≥250 | 9/74  119/702  32/188  19/97 | 12.2  17.0  17.0  19.6 | 0.64 |
| Assisted or breech delivery among all vaginal births | <50.0  50–149  150–249  ≥250 | 17/65  139/583  35/156  22/78 | 26.2  23.8  22.4  28.2 | 0.77 |
| Small for gestational age ^3^ | <50.0  50–149  150–249  ≥250 | 8/74  68/716  16/193  12/99 | 10.8  9.5  8.3  12.1 | 0.74 |
| Large for gestational age ^3^ | <50.0  50–149  150–249  ≥250 | 4/74  81/716  15/193  10/99 | 5.4  11.3  7.8  10.1 | 0.25 |

1. Values are number affected/total in the category (n/N). 2. *p* value from Chi-square test.
3. customized for maternal BMI, parity, sex and gestational age [34].

**Table S3.** Comparison of urinary iodine-to-creatinine ratio (UI/Creat; µg/g) in the first trimester only in those where pregnancy complications were either present or absent.

|  | **Median Urinary Iodine-to-Creatinine Ratio (µg/g) ^1^** | | | | ***p* Value ^2^** |
| --- | --- | --- | --- | --- | --- |
|  | ***n*** | **Complication Present** | ***n*** | **Complication Absent** |  |
| **Hypertensive disorders of pregnancy** | 264 | 110 (74–163) | 1455 | 107 (74–159) | 0.56 |
| *Pre-eclampsia* | 34 | 111 (81–148) | 1685 | 108 (74–160) | 0.86 |
| *Non-proteinuric gestational hypertension* | 230 | 109 (73–167) | 1489 | 108 (74–159) | 0.69 |
| **Any glucose derangement ^2^** | 122 | 106 (74–155) | 947 | 109 (74–155) | 0.88 |
| *Gestational diabetes* | 7 | 107 (62–173) | 1664 | 108 (74–160) | 0.69 |
| *Glycosuria (2+ or more)* | 53 | 125 (77–169) | 1666 | 107 (74–159) | 0.38 |
| *Hyperglycaemia during pregnancy* | 115 | 106 (74–154) | 981 | 109 (74–155) | 0.98 |
| **Anaemia during pregnancy** | 110 | 113 (85–158) | 986 | 107 (73–155) | 0.11 |
| **Anaemia <14 days postpartum** | 204 | 106 (77–165) | 1514 | 108 (74–159) | 0.68 |
| **Postpartum haemorrhage** | 154 | 106 (75–161) | 923 | 109 (74–153) | 0.95 |
| **Preterm delivery** | 79 | 102 (72–171) | 1648 | 108 (74–159) | 0.62 |
| **Mode of delivery** | **Caesarean** **section** | | **Vaginal delivery** | |  |
|  | 179 | 111 (77–161) | 882 | 107 (73–153) | 0.34 |
| **Type of vaginal delivery** | **Assisted/breech** | | **Spontaneous** | |  |
|  | 213 | 111 (72–155) | 669 | 105 (72–155) | 0.63 |
| **Customised birthweight ^3^** | **Small for gestational age** | | **Adequately grown for gestational age** | |  |
|  | 104 | 102 (65–158) | 868 | 110 (74–156) | 0.89 |
|  | **Large** **for gestational age** | | **Adequately grown for gestational age** | |  |
|  | 110 | 100 (76–145) | 868 | 110 (74–156) | 0.61 |

1. Values are median (25th, 75th percentile). 2. *p* value from Mann-Whitney U test. 3. customized for maternal BMI, parity, sex and gestational age [34].
